# Bias due to differential participation in case-control studies and review of available approaches for adjustment

**DOI:** 10.1371/journal.pone.0191327

**Published:** 2018-01-24

**Authors:** Annette Aigner, Ulrike Grittner, Heiko Becher

**Affiliations:** 1 Institute of Medical Biometry and Epidemiology, University Medical Center Hamburg-Eppendorf, Hamburg, Germany; 2 Center for Stroke Research, Charité–Universitätsmedizin Berlin, Berlin, Germany; 3 Department of Biostatistics and Clinical Epidemiology, Charité–Universitätsmedizin Berlin, Berlin, Germany; Centers for Disease Control and Prevention, UNITED STATES

## Abstract

**Objectives:**

Low response rates in epidemiologic research potentially lead to the recruitment of a non-representative sample of controls in case-control studies. Problems in the unbiased estimation of odds ratios arise when characteristics causing the probability of participation are associated with exposure and outcome. This is a specific setting of selection bias and a realistic hazard in many case-control studies. This paper formally describes the problem and shows its potential extent, reviews existing approaches for bias adjustment applicable under certain conditions, compares and applies them.

**Methods:**

We focus on two scenarios: a characteristic *C* causing differential participation of controls is linked to the outcome through its association with risk factor *E* (scenario I), and *C* is additionally a genuine risk factor itself (scenario II). We further assume external data sources are available which provide an unbiased estimate of *C* in the underlying population. Given these scenarios, we (i) review available approaches and their performance in the setting of bias due to differential participation; (ii) describe two existing approaches to correct for the bias in both scenarios in more detail; (iii) present the magnitude of the resulting bias by simulation if the selection of a non-representative sample is ignored; and (iv) demonstrate the approaches’ application via data from a case-control study on stroke.

**Findings:**

The bias of the effect measure for variable *E* in scenario I and *C* in scenario II can be large and should therefore be adjusted for in any analysis. It is positively associated with the difference in response rates between groups of the characteristic causing differential participation, and inversely associated with the total response rate in the controls. Adjustment in a standard logistic regression framework is possible in both scenarios if the population distribution of the characteristic causing differential participation is known or can be approximated well.

## Introduction

### Response rates

The advance in epidemiologic research depends on the willingness of contacted individuals to participate voluntarily in research. However, over the past decades this willingness to respond to research inquiries has substantially decreased,[1] which is reported as the proportion of responding out of all contacted individuals and most commonly referred to as the ‘response rate’. This also rendered it more difficult to acquire a random sample of controls free of a specific disease from the general population to match or compare to cases with the disease. This is the established approach in case-control studies, which is in turn still the currently most often used study design.

We know that this decrease in response rates has not necessarily been uniform over all groups of the population. Women are more likely than men to respond to research inquiries, just as individuals with a higher socioeconomic status, a higher education, and a current employment. On the other hand, there is no such clear trend for age and ethnicity.[1]

### Non-representative selection of controls

As in any empirical research, there are two main sources of error in epidemiologic studies: random error and systematic error. Whereas the first can be quantified using confidence intervals and reduces with increasing sample size, the systematic error is often overlooked.[2]

A specific type of systematic error is selection bias, which generally means that the association between outcome and exposure differs between selected individuals and all contacted or eligible individuals.[3] In epidemiology, selection bias has many labels depending on the setting, e.g. healthy-worker or non-response bias. However, all of them refer to the situation that the selection process of participants results in a non-representative sample with regard to the population of interest, and Hernan et al [3] show that all of them have the same underlying structure, which is the conditioning on a common effect.

We will further refer to the selection bias setting which we focus on, with differential participation bias. This term best describes the problem in research we want to address, which is the non-uniform willingness to participate as controls in a case-control study. The terms differential and non-differential are often used in the context of misclassification. In our context we use the term non-differential participation to refer to situations where the probability of participation is independent of covariates, and differential participation where the probability of participation differs between strata, defined by one or more, measured or unmeasured, covariates.

Issues for subsequent data analyses arise when the population characteristic causing the (differential) participation of controls is in turn linked to the outcome of main interest of a study. This can either be the case when this characteristic is associated with a risk factor, which is an example of selection bias only, or when the same characteristic is a genuine risk factor, which is an example of both selection bias and confounding.[3] Without controlling for this bias, the effect measures’ validity might be threatened.

### Adjustment by analysis

Knowledge about the potential presence of differential participation can help to reduce bias by design. In the setting of an epidemiologic study this can be done in two ways. First, knowing the true distribution of the characteristic causing differential participation, one can over-sample those for which the response rate is lower until the population distribution has been obtained. Second, if the true distribution is not known but a differential participation is assumed, one could ask those who refuse to participate a follow-up question about this specific characteristic only–to acquire information on the distribution, such that the sample can afterwards be weighted accordingly.[4] However, these approaches by design require researchers to know a priori which characteristic will cause differential participation in their specific field. Moreover they might need assumptions regarding this characteristic’s distribution, are limited to focus on one or only few such characteristics, and might require that participants answer to short follow-up questions, which could also be refused.

Therefore the approaches we focus on are based on the more common setting where an oversampling by design has not been performed, but valid external data sources are available to provide information on the true distribution of the characteristic causing differential participation. Thus it is still required that researchers have a notion of which such characteristics are potentially relevant in their field. However, the influence of several characteristics could be evaluated a posteriori, without implications to design, and therefore possibly less cost-intensive–given external data sources are available. A good example is a study focusing on mental health, where unemployment is considered a risk factor,[5] which is in turn associated with a lower participation rate.[1] In this case answering the sensitive follow-up question to estimate the population distribution of unemployment is likely to be refused, and it might not be the only characteristic causing differential participation, but valid external data is available.

The approaches we review do not employ probabilistic modelling of the selection bias (see e.g. Lash, Fox and Fink [2] for an introduction to probabilistic bias analysis), and are aimed at the application in a setting where a multidimensional systematic bias adjustment is necessary, i.e. where there are selection bias and confounding present at the same time. Lash, Fox and Fink [2] propose an adjustment approach for selection bias based on a similar approach, but in a setting where the exposure of interest causes differential participation and with known and probabilistically modelled selection probabilities for disease-exposure status, i.e. they weight the crude odds ratio (OR) based on the OR for the selection, but not individual observations. Although it is not common in practice to do so, one could include all contacted controls in the analyses, and therefore view the problem as a special case of missing data. However, this is not a useful option as we would then generally, unless implemented otherwise by design, have no information about them. In this setting, imputation techniques such as multiple imputation are not an option, as they need to draw from information in non-missing variables. A similar problem arises with inverse probability weighting if it is used to model the probability of missingness of each individual.[6]

Given the assumption that valid external data sources are available to provide information on the distribution of the characteristic causing differential participation, we first summarize available approaches and review their performance in two scenarios of this specific setting of bias due to differential participation, and further describe in more detail those approaches which are applicable in both scenarios and easily implementable in multivariable models. Second, the magnitude of the resulting bias on the targeted risk measure, the OR for case-control studies, is demonstrated under realistic scenarios. Finally, as an illustrative data example we use data from a case-control study on stroke where the response rate depended on an educational variable for which reference data was available.

## Materials and methods

### Problem statement

We denote the presence of a disease with *D* = 1 (e.g. stroke), and its absence with *D* = 0, and consider two binary characteristics–the exposure *E* (e.g. current smoking) and the covariate *C* (e.g. middle or low education), where the data can be presented in a 2x2 table *D* x *E*, stratified by levels of *C* (Table 1).

**Table 1:** 2x2 table disease *D* x exposure *E*, by levels of covariate *C*.

*C* = 1			*C = 0*	
	*D* = 1	*D* = 0	*D* = 1	*D* = 0
*E* = 1	a_1_	b_1_	a_2_	b_2_
*E* = 0	c_1_	d_1_	c_2_	d_2_

**Fig 1:**
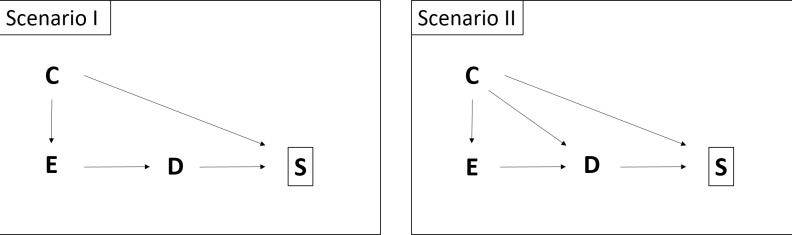
General graphical representation of Scenarios I & II. In both scenarios covariate *C* determines the response rate of the controls (selection for participation in study *S*); Scenario I: Covariate *C* is no confounder for the association of interest between exposure *E* and outcome *D*; Scenario II: *C* is a confounder.

We further consider two simple scenarios in both of which *C* determines the response rate, meaning that in different strata of *C* the response rates differ (Fig 1). In the first scenario, *C* is assumed to be linked to *D* only through its association with *E*, i.e. it is not a risk factor for *D* and therefore not a confounder. In this scenario, as *C* is not a risk factor for *D*, it is not needed in the analysis of the association between *E* and *D*. In the second scenario, we assume that *C* is a risk factor for *D* and associated with *E*, i.e. *C* is a potential confounder. It must be included in the model in order to obtain an unbiased estimate of the OR of *E* (OR_E_).

For both scenarios, we assume that (i) *E* is a risk factor without effect modification by *C*, i.e. OR_E|C = 1_ = (a_1_ d_1_) / (b_1_ c_1_) = OR_E|C = 0_ = (a_2_ d_2_) / (b_2_ c_2_) > 1, and (ii) *C* and *E* are positively associated.

The crude OR of *E* is OR_E_ = (a d) / (b c) = ((a_1_+ a_2_)(d_1_+ d_2_)) / ((b_1_+ b_2_)(c_1_+ c_2_)). We now assume a differential participation in controls by levels of *C*, indicated by the arrow from *C* to *S* in Fig 1. As a_1_, a_2_, c_1_, c_2_ relate to the case of *D* = 1, assuming differential participation in the controls, the only change is in the term (d_1_+ d_2_) / (b_1_+ b_2_). Let p_1_ be the response rate in controls when *C* = 1, p_0_ the response rate when *C* = 0, and p_1_ < p_0_. Then the relevant term changes accordingly and it follows (p_1_ d_1_ + p_0_ d_2_) / (p_1_ b_1_ + p_0_ b_2_) > (d_1_ +d_2_) / (b_1_ + b_2_), i.e. OR_E_ increases and is biased. Given the example of a crude OR_E_ of 2.25, a differential response rate p_1_ of 0.2 and p_0_ of 0.8 results in a biased OR_E_ of 2.70 (see S1 Table for this numerical example).

### Review of available approaches for bias adjustment due to differential participation

There are different approaches to adjust for the differential participation bias in the OR estimates, where their applicability depends on the specific setting at hand.

#### Adjusting the OR_E_ estimate

OR_E_ can be adjusted either

(i) by the inclusion of the variable *C* as a covariate in a logistic regression model,[7] or
(ii) by using a stratified analysis by strata of *C* (e.g. conditional logistic regression conditioning on *C*,[8] Cochran-Mantel-Haenszel-statistic[9, 10]).

It should be noted here that models including *C* as a covariate are fundamentally different from models not including *C*, and that these different models are not simply comparable. Yet, we still included the model with *C* as a covariate in scenario I for illustrative reasons. Moreover, if valid reference data is available for the distribution of *C*, the over- or underrepresentation by strata of *C* can also be incorporated

(iii) by the use of an appropriate offset term in logistic regression, or
(iv) by weighting observations in logistic regression,

as summarized in Table 2. It has to be emphasized here that the use of weights or an offset might aggravate the problem of heteroscedasticity, and therefore valid inferences for these models require a robust estimation of the standard error.

**Table 2:** Overview models and adjustment approaches for Scenarios I & II.

Scenario	Adjustment	Model	Unbiased estimate of	
			OR_E_	OR_C_
I	None (basic model)	logit(Y = 1|E) = α + β_1_ e	no	-
	Variable inclusion	logit(Y = 1|E,C) = α + β_1_ e + β_2_ c	yes	no
	Stratification	logit(Y = 1|E) = α + β_1C0_ e logit(Y = 1|E) = α + β_1C1_ e combine β_1C0_ and β_1C1_, e.g. with Cochran-Mantel-Haenszel-statistic	yes	-
	Weights^a^	logit(Y = 1|E) = α + β_1_e using weighted observations	yes	-
	Offset^b^	logit(Y = 1|E) = α + offset + β_1_ e	yes	-
II	None (basic model)	logit(Y = 1|E,C) = α + β_1_ e + β_2_ c	yes	no
	Weights^a^	logit(Y = 1|E,C) = α + β_1_ e + β_2_ c using weighted observations	yes	yes
	Offset^b^	logit(Y = 1|E,C) = α + offset + β_1_ e + β_2_ c	yes	yes

^a^ see paragraph *Adjustment by weights*

^b^ see paragraph *Adjustment by offset*

#### Adjusting the OR_C_ estimate

In both scenarios, the simple inclusion of *C* in the model will lead to a biased estimate of OR_C_. To also derive an unbiased estimate of OR_C_, we have to make use of reference data, as suggested in approaches (iii) and (iv).

Both of these two approaches require external data sources, but only they are applicable in both scenarios and can be used to derive both estimates, OR_E_ and OR_C_, and are therefore introduced in more detail subsequently. However as the other approaches also yield valid results for OR_E_, they will be further included in simulations and the study example in scenario I.

#### Adjustment by weights

If we knew the correct values for response rates given *C* = 1 and *C* = 0, i.e. p_1_ and p_0_, we could adjust the relevant quantities with the inverse terms 1/p_1_ and 1/p_0_, respectively, which can also be referred to as weighting the data affected by differential participation.

If these quantities are not known, they can be estimated given knowledge about the true distribution of *C* in the general (assumed healthy) population via reference data. We compare the proportion of *C* in the sample of controls to its proportion in the population, i.e. define two scalars as fractions of proportions: $\hat{p_{1}}$ = (sample proportion *C* = 1) / (population proportion of *C* = 1), and $\hat{p_{0}}$ = (1 –sample proportion *C* = 1) / (1 –population proportion of *C* = 1).

These proportions indicate whether controls with *C* = 1 or *C* = 0 are over- or underrepresented in the observed sample, and to what extent. In the case of a logistic regression model, we would then weight each observation *i* as follows:

$${weight}_{i}=\left\{ \begin{array}{l} (1/\hat{p_{1}}),\;if\;i\;is\;a\;control\;with\;C=1\\ (1/\hat{p_{0}}),\;if\;i\;is\;a\;control\;with\;C=0\\ \;1,\;if\;i\;is\;a\;case, \end{array}\right.$$

such that a case has the standard weight of 1, a control with its level of *C* being underrepresented obtains a weight > 1, and vice versa. Scott and Wild [11] discuss introducing individual weights in maximum likelihood estimation. This idea corresponds to the idea of inverse probability weighting to correct for unequal sampling fractions,[6] like the Horvitz-Thompson estimate.[12] The weights proposed above can be rewritten as $\frac{1}{\frac{P(C=i|S)}{P(C=i)}}$, where *P(C* = *i*|*S)* is the sample proportion of *C* = *i*, and *P(C* = *i)* is the population proportion of *C* = *i*, given by external data. Due to Bayes theorem these weights are equivalent to $\frac{1}{\frac{P(S|C=i)}{P(S)}}$. As the sampling fraction *P(S)* is a constant, this expression is proportional to the Horvitz-Thompson estimate, where weights are estimated by $\frac{1}{P(S|C=i)}$.

#### Adjustment by offset

Breslow and Cain [13] and Cain and Breslow [14] showed that the introduction of an offset to the logistic regression model can account for bias. Although they applied this approach to a different setting, the fundamental problem is mathematically equivalent. They assume that the risk factor of main interest *E* is sampled in the total study population in a first stage, and the potential confounder *C* is sampled only in a subset of the non-diseased, with predefined selection probabilities for each category of *E* (choice-based sampling), such that a complete case analysis would lead to a biased estimate of the OR_E_. They showed that an unbiased OR estimate for E can be obtained if the offset is subsequently defined as the logarithm of the ratio of the selection probabilities in the strata. This situation is equivalent to the present situation where *C* is known for the total population and *E* is known for a sample of the controls which is not representative for *C*, where we define the offset for each observation *i* as follows:

$${of\;f\;set}_{i}=\left\{ \begin{array}{l} \ln\;(\hat{p_{0}}/\hat{p_{1}}),\;if\;C=1\\ \;0,\;otherwise, \end{array}\right.$$

where $\hat{p_{0}}/\hat{p_{1}}$ is the OR of *C* for controls vs. population.

### Data

#### Simulation

In order to demonstrate the magnitude of the resulting bias if the differential response is ignored, and to illustrate the performance of the mentioned approaches for adjustment, we simulate a case-control study.

In a first simulation step, we simulate a large cohort with N = 3,000,000 individuals under specific assumptions for the binary variables *C*, *E*, and *D* (Table 3) for both previously introduced scenarios (Fig 1). In a second simulation step, based on assumptions regarding participation probabilities by strata of *C*, we set certain proportions of the controls missing, (e.g. 40% if *C* = 0 and 70% if *C* = 1) and sample 1,000 individuals from the diseased (cases) and 2,000 from the non-diseased (controls). We replicate this step 1,000 times and calculate the OR estimate according to the various approaches.

**Table 3:** Simulation parameters for Scenario I & II of the association between outcome *D*, exposure *E*, and covariate *C*.

	Scenario I	Scenario II
Prevalence of *C*	P(*C* = 1) = 0.5	
Prevalence of *E* conditioned on *C*	P(*E* = 1 | *C* = 0) = 0.2 P(*E* = 1 | *C* = 1) = 0.4 → P(*E*) = 0.3	
Prevalence of *D* conditioned on *E* and *C*	P(*D* = 1|*E* = 0, *C*) = 0.05 P(*D* = 1|*E* = 1, *C*) = 0.1	P(*D* = 1|*E* = 0, *C* = 0) = 0.05 P(*D* = 1|*E* = 0, *C* = 1) = 0.1 P(*D* = 1|*E* = 1, *C* = 0) = 0.1 P(*D* = 1|*E* = 1, *C* = 1) = 0.2
Prevalence of *D*	6.5%	10.1%
Crude odds ratio of *E*	2.1	2.2
Crude odds ratio of *C*	1.2	2.2

In all analyses, we consider the OR estimates for *C* and *E* derived from logistic regression analysis as the target parameters. For graphic representation of the simulation results we use 10% as an arbitrary threshold for substantial difference between true and biased effect.

For all analyses the statistical software R was used,[15] the R package epiR for the Cochran-Mantel-Haenszel OR estimate,[16] the packages lmtest [17] and sandwich [18, 19] for robust estimates of the standard error.

#### Study example

**Fig 2:**
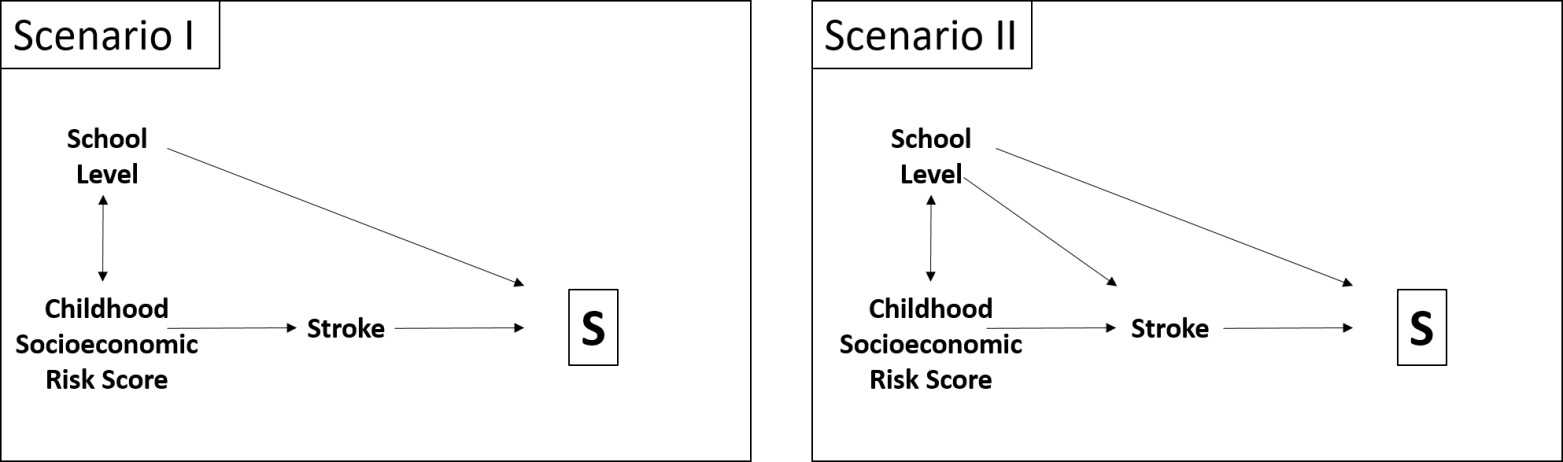
Graphical representation of Scenarios I & II for the study example. In both scenarios the covariate school level determines the response rate of the controls (selection for participation in study *S*); Scenario I: School level is no confounder for the association of interest between childhood socioeconomic risk score and stroke; Scenario II: School level is a confounder.

The application of this approach is demonstrated via data from the GENESIS study (Inflammatory, Genetic and Socio-economic Determinants of Ischemic Stroke and their Interdependence), a case-control study on stroke. The study was approved by the ethics committee of the Landesärztekammer Rheinland-Pfalz (837.333.05(4991)). It was carried out in Ludwigshafen, Germany, and includes data on 470 stroke patients and 809 age- and sex frequency-matched controls. One main hypothesis of this study was that adverse socioeconomic conditions in childhood (living, familial, material, and self-estimated financial conditions during childhood up to age 14), adolescence, and adulthood each independently contribute to the risk of first-ever ischemic stroke.[20] As an example, we will focus on the results for the childhood socioeconomic risk score (corresponding to *E* in Fig 1). The socioeconomic risk score in childhood ranged from 0 (low) to 10 (high) and was divided into three levels (low, middle, high). The controls were randomly selected from the total population of Ludwigshafen. About 50% of the initially contacted individuals were recruited, which raised awareness of potential response bias in the controls, known to often depend on the educational level (corresponding to *C* in Fig 1), which is in turn positively associated with the socioeconomic score of a respondent (Fig 2).

We obtained reference data for the distribution of highest obtained educational level in the controls, by age and sex groups for the general population from the statistical office of the city of Ludwigshafen (Stadtverwaltung Ludwigshafen) for the years 1970 and 1987 (Table 4).

**Table 4:** Study data of the GENESIS study and reference data for the general population of ludwigshafen by sex and age.

Sex	Age	Study Data			Ludwigshafen Population		
		School Level %			School Level %		
		Low	Middle	High	Low	Middle	High
Female	<60	42.5	33.3	24.1	59.0	25.1	15.9
	61–70	73.9	21.6	4.6	72.8	18.0	9.2
	71+	68.7	22.1	9.2	78.2	15.1	6.7
Male	<60	50.6	25.9	23.5	58.5	17.7	23.8
	61–70	64.5	20.7	14.8	73.2	12.9	13.9
	71+	68.1	17.0	14.9	79.0	10.9	10.2

Source: Statistical Office (Stadtverwaltung) Ludwigshafen: Evaluation of the population census 1970 and 1987. Highest achieved school education (1970) and highest professional training (1987) by age and sex groups.

## Results

### Simulation

#### Scenario I

**Fig 3:**
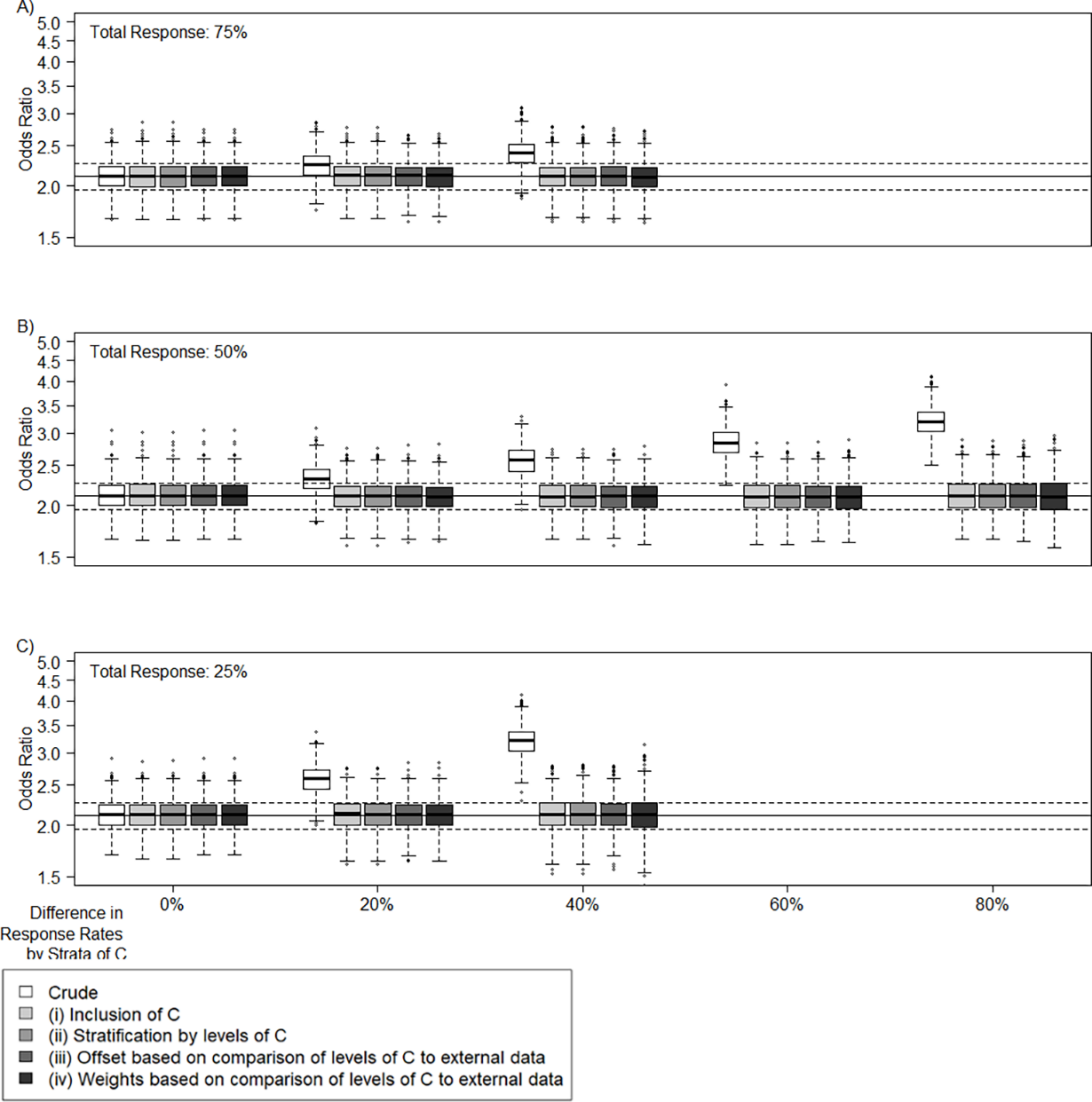
Simulations Scenario I for different total response rates and differences in response rates by strata of *C*. Crude and differential participation adjusted odds ratios (OR) of exposure *E* (y-axis, logarithmic scale) with regard to the disease *D* (true OR = 2.1); Solid lines indicate unbiased OR; dashed lines indicate 10% margins of unbiased OR; Simulations based on scenario I (*C* is a covariate but no confounder, *C* determines the response rate), different total response rates (A: 75%, B: 50%, C: 25%), and differences in response rates by strata of *C* (x-axis, no difference means same response rate in each strata of *C*) (1000 cases, 2000 controls, 1000 simulations); The larger the difference in response rates by strata of *C* and the lower the mean response rate, the higher the bias in the crude OR.

The distribution of the resulting crude and adjusted OR_E_ estimates with all proposed approaches based on various settings of participation probability are displayed as boxplots (Fig 3). The true OR estimate, i.e. given a probability of participation of 100% in both groups of *C*, is indicated with the black line. This estimate is the same for all settings of non-differential participation, indicated with the first boxplot, respectively (difference: 0%).

The bias of the crude OR is larger, the larger the differences between response rates in the strata of *C* and the lower the mean response rate. We observe a positive bias, as only settings are plotted where the high risk group (*C* = 1) is assumed to have a lower response. Assuming 10% from the true OR as an acceptable deviation (indicated with dashed lines), the biased OR estimates do not exceed these limits for minor differences in response rates and a rather high total response rate. However, in the majority of the displayed settings, the crude OR estimates lie beyond this range.

As expected, all proposed approaches for adjustment give an unbiased estimate of the OR_E_. Small differences in the variability of the results are visible, where especially the estimate adjusted with weights displays a higher variability (see S1 File for the R script).

Additional simulations were run assuming different ORs in this scenario, showing that the size of the effect does not influence the size of the bias (S1 Fig). We furthermore ran analyses for varying the matching rate for cases and controls, from cases:controls = 1:1 to 1:4, where we see that this matching rate does not impact the bias in the analysis, but only the variability in the estimate (S2 Fig).

#### Scenario II

**Fig 4:**
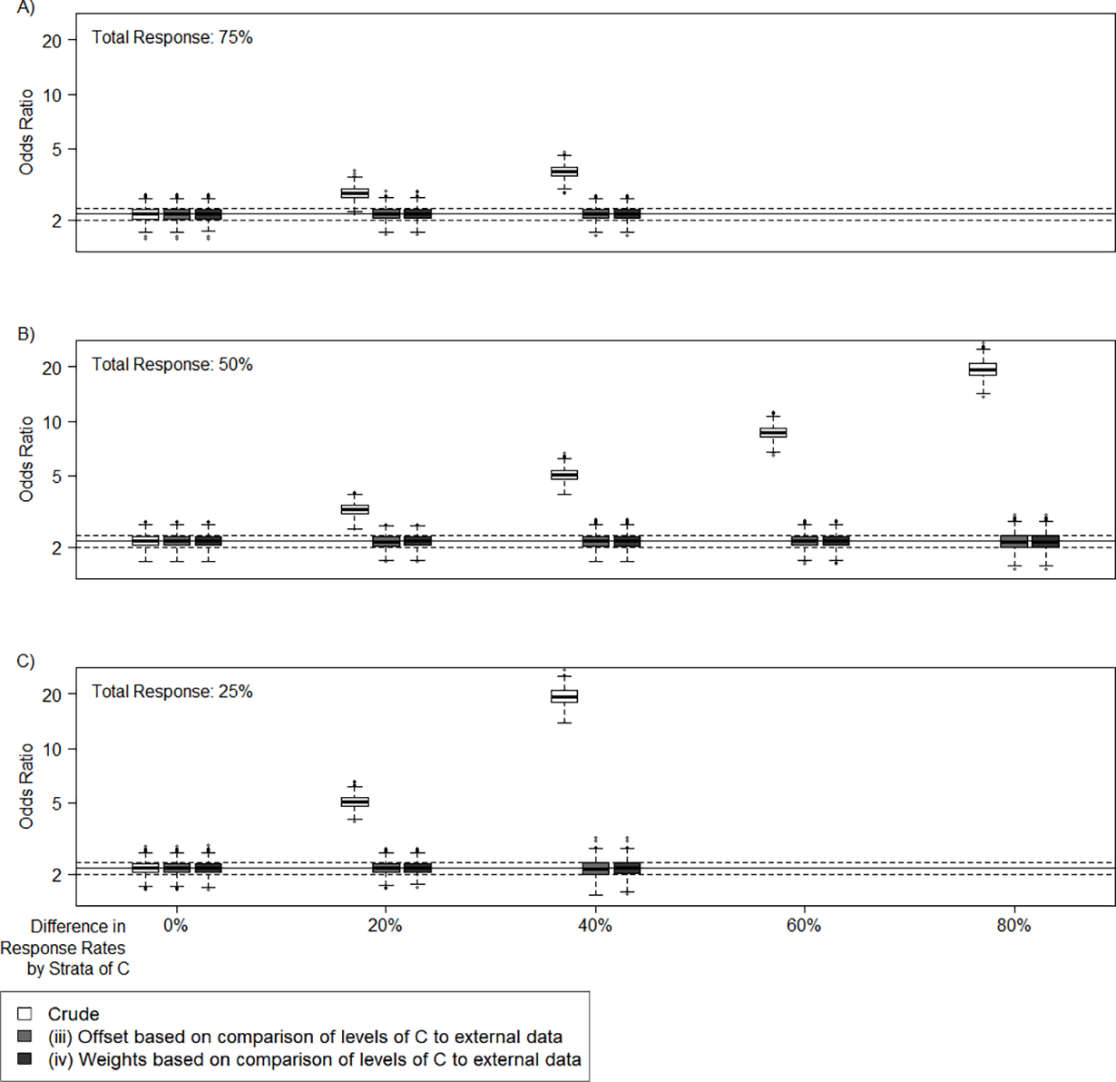
Simulations Scenario II for different total response rates and differences in response rates by strata of *C*. Crude and differential participation adjusted odds ratios (OR) of covariate *C* (y-axis, logarithmic scale) with regard to the disease *D* (true OR = 2.2); Solid lines indicate unbiased OR; dashed lines indicate 10% margins of unbiased OR; Simulations based on scenario II (*C* is a covariate and a confounder, *C* determines the response rate), different total response rates (A: 75%, B: 50%, C: 25%), and differences in response rates by strata of *C* (x-axis, no difference means same response rate in each strata of *C*) (1000 cases, 2000 controls, 1000 simulations); The larger the difference in response rates by strata of *C* and the lower the mean response rate, the higher the bias in the crude OR.

Assuming that both *E* and *C* are risk factors, Fig 4 presents boxplots of the expected OR_C_, based on logistic regression models including *E* and *C* as independent variables. The figure also displays the offset and weight adjusted OR_C_ estimates.

The bias for non-differential participation in this scenario is again 0 in expectation and again positive for all settings of differential participation. The vast majority of biased OR estimates due to differential participation exceed the 10% deviation limits, especially clearly assuming a low total participation rate (Fig 4). The bias due to differential participation in the OR_C_ estimate is also a lot more distinct compared to the bias in the OR_E_ in scenario I. The results demonstrate that unbiased effect estimates can be calculated even if *C* is a confounder in the association of *E* and *D* (see S2 File for the R script).

### Study example

The risk factor of main interest *E* in the example of the GENESIS study is the childhood socioeconomic risk score, and the characteristic *C* assumed to have caused differential participation is educational achievement measured with the highest school grade, which might additionally be a risk factor. The childhood socioeconomic risk score is positively associated with school level (Kendall’s tau = 0.29 and 0.31 in cases and controls, respectively), and as such we expect an overestimation of the ORs in both scenarios if differential participation is ignored (the minimal anonymized dataset is provided with figshare.com: https://figshare.com/articles/GENESIS_ReplicationDataset_csv/5580091; see S3 File for the R script reproducing Table 5).

**Table 5:** Odds ratio estimates and 95% confidence intervals for stroke risk factors of main interest in Scenario I & II. Scenario I: Only confounding adjusted, i.e. adjustment for: two-year age groups and sex; Scenario II: Confounding and differential participation adjusted; Analyses based on logistic regressions (n = 1279, events = 470).

Scenario	Variables	Adjustment Approach				
		None (basic model)	Variable inclusion	Stratification	Weights^a^	Offset^a^
I	Risk Score Childhood^b^: *Low*	1	1	1	1	1
	*Middle*	2.20(1.65–2.95)	1.92(1.42–2.62)	1.92(1.41–2.61)	1.93(1.43–2.62)	1.89(1.39–2.57)
	*High*	2.95(2.14–4.09)	2.55(1.82–3.59)	2.54(1.81–3.57)	2.61(1.87–3.64)	2.61(1.86–3.66)
II	Risk Score Childhood^b^: *Low*	1	-	-	1	1
	*Middle*	1.92(1.42–2.62)			1.93(1.41–2.64)	1.88(1.37–2.58)
	*High*	2.55(1.82–3.59)			2.61(1.85–3.68)	2.59(1.83–3.68)
	School Level: *High*	1	-	-	1	1
	*Middle*	0.62(0.39–0.99)			0.75(0.46–1.21)	0.76(0.47–1.24)
	*Low*	1.24(0.85–1.84)			0.89(0.59–1.34)	0.92(0.61–1.38)

^a^ based on reference data from the Statistical Office (Stadtverwaltung) Ludwigshafen (Table 4)

^b^ as defined in the original publication by Becher et al [20]: categories based on classifications of the summed risk scores, based on the distribution in controls; The score is based on information on the parental professions, living conditions, and estimated family income in childhood.

#### Scenario I

All four approaches can be used to obtain an unbiased estimate of the OR_E_ in scenario I. Weights and offsets are derived from the reference data for each school level-age-sex-group (Table 4). As an example, 24.1% of women under 60 obtained a high school level in our study data, and only 15.9% in the reference data, the weight for female controls under 60 with a high school level is therefore 1/(24.1/15.9) = 0.6598, smaller than 1 due to their overrepresentation in the controls.

All results are given in the upper part of Table 5. In this setting, where only one risk factor is of interest and we additionally control for confounding by age and sex only, all approaches yield highly similar results for OR_E_. The similarity between those estimates based on reference data and those of the other approaches speaks in favor of the reference data’s validity. The figures confirm our expectation of OR_E_ estimates decreasing towards 1, given the adjustment, where both OR_E_ estimates decrease by about 12 to 14%.

#### Scenario II

If the school level *C* itself is assumed to be a risk factor, we would like to obtain an unbiased estimate of OR_E_ and OR_C_ simultaneously, and therefore adjust with weights or an offset. Results are shown in the lower part of Table 5. The OR_E_ estimate is unbiased in expectation in all presented settings, even without further adjustment. However in this scenario we assume the OR_C_ being biased, as it also causes differential participation. We see that the crude OR_C_ estimate suggests a significant protective effect of a middle school level compared to a high school level, and an elevated risk for low school level, although not significant. Again, the weights and offset adjustment yield highly similar results, in a way that all OR estimates change towards 1 (for the second OR even below 1).

## Discussion

We showed that several approaches can be applied to derive unbiased effect measures in a specific setting of selection bias, where the response rate in controls depends on covariates. In cases where these covariates also act as independent risk factors, we can still derive their unbiased effect measures, but need reference data to be available as a valid estimate of the covariates’ distribution in the underlying population. The comparison of the population distribution to the study data's distribution is used to include weights or an offset term in a standard logistic regression framework.

Considering two scenarios of the situation that the characteristic causing differential participation is in turn linked to the outcome of main interest of a study–either by being correlated with a risk factor, or as a genuine risk factor itself, the extent of the bias in the OR estimation was investigated with simulations. These showed that the bias in the OR estimates is positively associated with the difference in response rates between groups of the characteristic causing participation, and inversely associated with the total response rate in the controls. The bias of the effect measure in both scenarios can be quite large. For example, in a study with a total response of 50% and a difference of 20% in response rates between groups of the characteristic causing participation, where this characteristic is itself a risk factor (scenario II), the OR was highly overestimated.

Both scenarios are realistic in many case-control study settings. The sex of the participating controls is a demonstrable example for the second scenario–we know from research on response rates that women are more likely to participate as controls in a study,[1] and that sex is often associated with the probability of disease occurrence, such as stroke, which is more likely to occur in men.[21] The effect estimate for sex will therefore be biased in a standard analysis. If, as common in practice, cases and controls are matched according to the variable causing differential participation, i.e. sex, this bias will not arise, but also no effect estimate for this variable can be derived. Many other plausible examples can be given for different fields of research. The current socioeconomic status has for example been shown to be independently associated with both stroke incidence and mortality,[20, 22] and may be a driving factor for differential participation;[1] Unemployment is an independent risk factor for mental health,[5] and at the same time associated with a lower participation in scientific studies;[1] Finally, marital status is associated with a lower cancer mortality,[23] and married individuals are more likely to respond to research inquiries.[1]

### Generalizations

We primarily considered here the simple case with two binary variables *E* and *C*, however, the approach can be extended to the more general case with any *k* exposures *E*_*1*_, …, *E*_*k*_ and any categorical variable for *C*, as shown here in the data example. It is furthermore not limited to a differential participation in the controls, the same framework can be applied in the presence of differential participation in the cases, which may only be a less common issue in the setting of a case-control study.

Loosening the assumption of valid reference data, one could either use at least an educated guess or a range of educated guesses, or use probability distribution assumptions for the reference data to make a probabilistic bias analysis.

### Limitations

Although the approach is easily extended to variables with more than two categories, the case of continuous variables needs further investigation, but the same approach is conceivable.

Independent of the analytical method, the use of reference data can be problematic, as the assumption of the data’s validity might not hold. In the study example, we make use of reference data which showed some degree of validity as the results from all approaches in scenario I were highly similar. However potential issues with the validity of the reference data persist, but were not further investigated in this study.

Adjustment with weights or an offset has two further disadvantages. First, the variance of the OR estimates increases taking the adjustment into account, even more so given a high variation in weights/offset. Second, the approaches are sensitive to outliers, especially in settings with small numbers of observations for certain strata.[24, 25]

## Conclusions

Our study aimed to improve the analysis of epidemiologic studies by pointing out a potential source of bias and its possible extent, and to advocate the implementation of approaches for bias adjustment. The importance of doing so was previously emphasized by Lash et al.[26]

Therefore we illustrated the extent of the bias introduced by differential participation in a case-control setting, and reviewed existing approaches for bias adjustment, which are easily implementable in standard statistical software in the common setting of multivariable models, where some depend on the assumption of valid reference data being available.

## Supporting information

S1 Table Numerical example of 2x2 table disease D x exposure E, by levels of covariate C. (DOCX)

S1 Fig Simulations Scenario I based on different odds ratios. Crude and differential participation adjusted odds ratios (OR) of exposure *E* (y-axis, logarithmic scale) with regard to the disease *D* (true OR = 1.3, 2.1, 3.4); Simulations based on Scenario I (*C* is a covariate but no confounder, *C* determines the response rate), total response rate of 40%, and difference in response rates of 40% by strata of *C* (x-axis) (n = 1000 cases, 2000 controls, 1000 simulations); The bias in the crude OR is not affected by the effect size.
(TIFF)

S2 Fig Simulations Scenario I based on different matching rates cases:Controls. Crude and differential participation adjusted odds ratios (OR) of exposure *E* (y-axis, logarithmic scale) with regard to the disease *D* (true OR = 1.3, 2.1, 3.4) based on different matching rates cases:controls (1:1, 1:2, 1:3, 1:4); Simulations based on Scenario I (*C* is a covariate but no confounder, *C* determines the response rate), total response rate of 40%, and difference in response rates of 40% by strata of *C* (x-axis) (n = 1000 cases, 1000 simulations); The higher the number of controls, the lower the variation in the estimate, the bias in the crude OR is not affected.
(TIFF)

S1 File R Script simulation code for differential response bias in Scenario I. (R)

S2 File R Script simulation code for differential response bias in Scenario II. (R)

S3 File R Script based on GENESIS study replication dataset for Table 5. (R)
