# Supplementary material for: Bias due to differential participation in case-control studies and review of available approaches for adjustment
Source: PLoS One. 2018 Jan 24;13(1):e0191327. doi: 10.1371/journal.pone.0191327 (PMC5783376; doi:10.1371/journal.pone.0191327)
Supplement: S1 Table — (DOCX) [file pone.0191327.s001.docx]

| *C* = 1 | | | *C = 0* | |
| --- | --- | --- | --- | --- |
|  | *D* = 1 | *D* = 0 | *D* = 1 | *D* = 0 |
| *E* = 1 | 20 | 10 | 10 | 10 |
| *E* = 0 | 10 | 10 | 10 | 20 |

OR_E|C=1_ = (a_1_ d_1_) / (b_1_ c_1_) = OR_E|C=0_ = (a_2_ d_2_) / (b_2_ c_2_) = 200/100 = 2

crude OR of *E* = (a d) / (b c) = ((a_1_+ a_2_)(d_1_+ d_2_)) / ((b_1_+ b_2_)(c_1_+ c_2_))

= (30*30)/(20*20) = 2.25

p_1_ … response rate in controls when *C* = 1, p_0_ … response rate when *C* = 0

e.g. p_1_ = 0.2 < p_0_ = 0.8

OR_E_ = ((a_1_+ a_2_)( p_1_ d_1_+ p_0_ d_2_)) / ((p_1_ b_1_+ p_0_ b_2_)(c_1_+ c_2_))

= ((20+ 10)*( 0.2*10 + 0.8*20)) / ((0.2*10 + 0.8*10)*(10 + 10)) = 2.7
