# Supplementary figures and images for: Bias due to differential participation in case-control studies and review of available approaches for adjustment
Source: PLoS One. 2018 Jan 24;13(1):e0191327. doi: 10.1371/journal.pone.0191327 (PMC5783376; doi:10.1371/journal.pone.0191327)

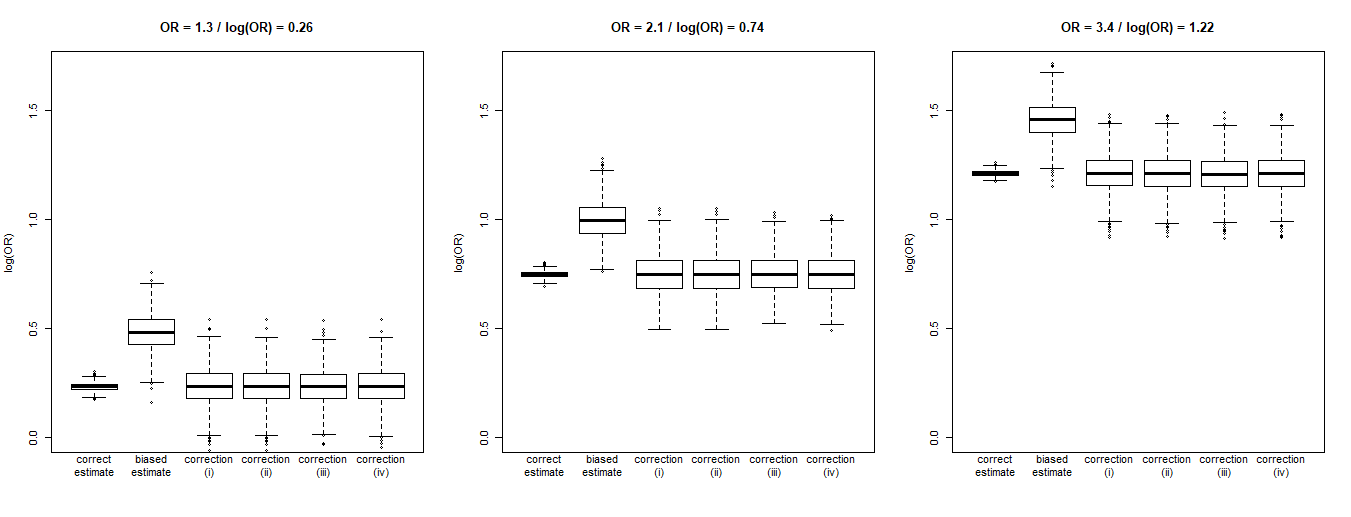

Supplement: S1 Fig — Crude and differential participation adjusted odds ratios (OR) of exposure E (y-axis, logarithmic scale) with regard to the disease D (true OR = 1.3, 2.1, 3.4); Simulations based on Scenario I (C is a covariate but no confounder, C determines the response rate), total response rate of 40%, and difference in response rates of 40% by strata of C (x-axis) (n = 1000 cases, 2000 controls, 1000 simulations); The bias in the crude OR is not affected by the effect size. (TIFF) [file pone.0191327.s002.tiff]

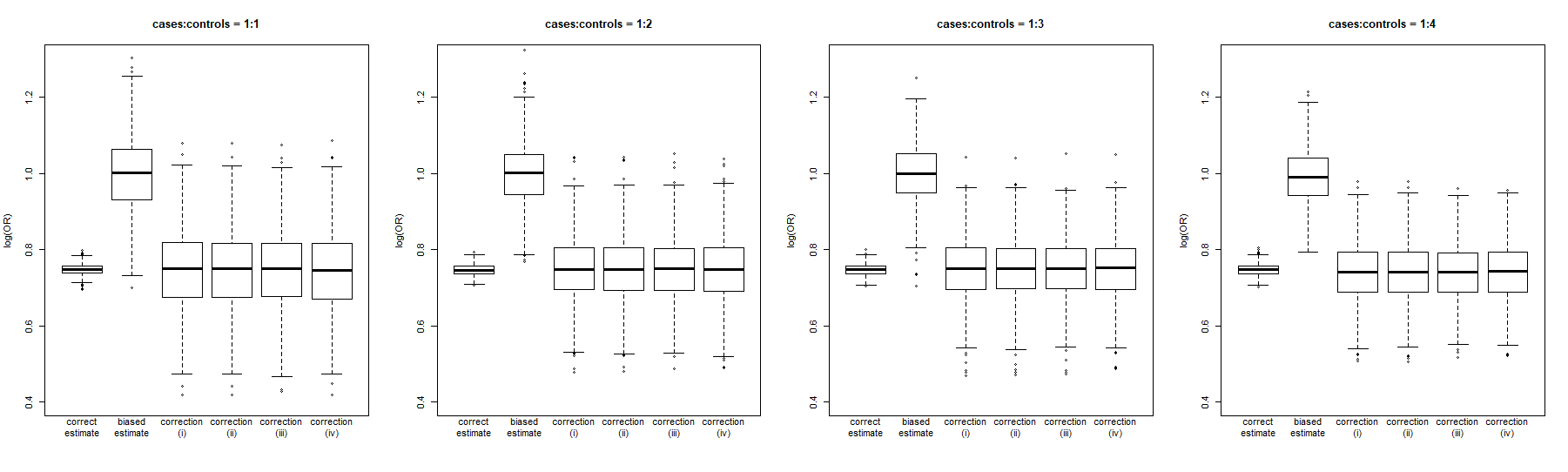

Supplement: S2 Fig — Crude and differential participation adjusted odds ratios (OR) of exposure E (y-axis, logarithmic scale) with regard to the disease D (true OR = 1.3, 2.1, 3.4) based on different matching rates cases:controls (1:1, 1:2, 1:3, 1:4); Simulations based on Scenario I (C is a covariate but no confounder, C determines the response rate), total response rate of 40%, and difference in response rates of 40% by strata of C (x-axis) (n = 1000 cases, 1000 simulations); The higher the number of controls, the lower the variation in the estimate, the bias in the crude OR is not affected. (TIFF) [file pone.0191327.s003.tiff]
